# Clinical Significance and Management of Atrioventricular Block Associated With Bradycardic/Antiarrhythmic Drug Therapy: Drug‐Induced or Drug‐Revealed?

**DOI:** 10.1111/jce.16697

**Published:** 2025-04-28

**Authors:** Dimitrios Sfairopoulos, George Bazoukis, Skevos Sideris, Nikolaos Fragakis, Konstantinos Letsas, Konstantinos Zekios, Tong Liu, Panagiotis Korantzopoulos

**Affiliations:** ^1^ First Department of Cardiology University of Ioannina Medical School Ioannina Greece; ^2^ Department of Cardiology Larnaca General Hospital, State Health Services Organization Larnaca Cyprus; ^3^ Department of Basic and Clinical Sciences University of Nicosia, Medical School Nicosia Cyprus; ^4^ State Department of Cardiology “Hippokration” General Hospital of Athens Athens Greece; ^5^ Second Department of Cardiology Aristotle University of Thessaloniki, Hippokration General Hospital Thessaloniki Greece; ^6^ Arrhythmia Unit Onassis Cardiac Surgery Center Athens Greece; ^7^ Tianjin Key Laboratory of Ionic‐Molecular Function of Cardiovascular Disease, Department of Cardiology, Tianjin Institute of Cardiology Second Hospital of Tianjin Medical University Tianjin China

**Keywords:** antiarrhythmic drugs, atrioventricular block, digoxin, hyperkalemia, nondihydropyridine calcium channel blockers, pacing, β‐blockers

## Abstract

The development of advanced atrioventricular block (AVB) in patients on bradycardic and/or antiarrhythmic therapy (drug‐related AVB) represents a clinical challenge, raising the question of whether the AVB is directly caused by these agents (drug‐induced AVB) or if the offending drugs exacerbate an underlying conduction system disease. Traditionally, β‐blockers, non‐dihydropyridine calcium channel blockers, class Ic/III antiarrhythmics, and digoxin have been considered reversible causes of advanced AVB. However, recent evidence shows a weak cause‐and‐effect relationship between these drugs and AVB in the elderly, along with high recurrence rates of AVB despite initial resolution after drug discontinuation. This may also apply to patients on high doses of these medications, drug combinations, or with additional reversible factors such as hyperkalemia. Despite these considerations, the European Guidelines do not suggest permanent pacing for AVB due to transient causes that are correctable, including bradycardic/antiarrhythmic drug therapy. On the other hand, the American Guidelines recommend permanent pacing for selected patients with symptomatic second‐ or third‐degree AVB who are on stable, necessary antiarrhythmic or β‐blocker treatment, without waiting for drug washout or reversibility. Notably, an accumulating body of evidence indicates that true drug‐induced AVB is rare, while recurrence rates are high. Therefore, early permanent pacing should be recommended, especially for frail elderly patients. Moreover, in patients with drug‐related AVB and atrial tachyarrhythmias, adopting an early permanent pacing approach seems prudent when bradycardic and/or antiarrhythmic treatment is necessary. Finally, delays in permanent pacing are not justified when temporary pacing is needed, given the increased associated risks in such cases.

## Graphical abstract

Schematic representation of the management pathway in drug‐related advanced atrioventricular block.

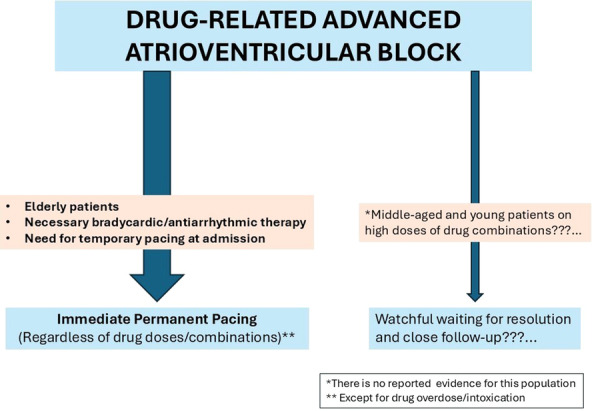

**Abbreviations**
AVB: atrioventricular block
CCBs: calcium channel blockers
non‐DHP: nondihydropyridine

## 1 Introduction

Atrioventricular block (AVB) that develops in patients receiving bradycardic and/or antiarrhythmic drug therapy (drug‐related AVB) presents a clinical challenge, prompting considerations about whether the AVB is directly induced by the medications (drug‐induced AVB) or if it reveals a pre‐existing, severe conduction system disease [1]. B‐blockers, nondihydropyridine calcium channel blockers (non‐DHP CCBs), class Ic/III antiarrhythmics, and digoxin have been considered reversible causes of AVB in clinical practice [2, 3, 4]. However, an accumulating body of evidence demonstrates a weak cause‐and‐effect relationship between treatment with these agents and AVB, as well as high recurrence rates of AVB following initial resolution after drug discontinuation [1, 5]. Therefore, there is a discrepancy between the traditional perception of the natural history and prognosis of patients with drug‐related AVB and the actual clinical outcomes observed in real‐life clinical situations.

Indeed, current guidelines reflect this ambiguity. The European Society of Cardiology Guidelines do not recommend permanent pacing for AVB due to transient factors that can be corrected and prevented (Class III recommendation, Level of Evidence C) [4]. On the other hand, the Guidelines of the American College of Cardiology/American Heart Association suggest that permanent pacemaker implantation may be reasonable for certain patients with symptomatic second‐ or third‐degree AVB who are on chronic stable doses of necessary antiarrhythmic or β‐blocker therapy without further observation for drug washout or reversibility (class IIa recommendation, level of evidence B) [6]. Additionally, they note that while a bradycardic drug overdose may cause reversible AVB, therapeutic doses of these medications typically do not lead to AVB [6]. Therefore, the management of drug‐related AVB remains a topic of debate.

In this context, we sought to synthesize current evidence on the clinical characteristics of drug‐related AVB, assess recovery and recurrence outcomes, and propose management strategies for these patients based on the available data reported in the literature.

## 2 Current Evidence on Drug‐Related Atrioventricular Block

The existing evidence regarding drug‐related AVB is predominantly drawn from observational studies. The subsequent section provides a detailed examination of the available published studies in this field, with an overview presented in Table 1 and Table S1.

**Table 1:** Studies examining the outcomes of patients with drug‐related AV block (AVB). Age, follow‐up duration, and time from inclusion to AVB recurrence are presented as mean ± SD or median [IQR].

First author, year	Study type, number of patients	Age (years), males (%)	Inclusion criteria, (%) of patients with each bradyarrhythmia	Exclusion criteria	Follow‐up period	Time to AVB/Bradyarrhythmia recurrence	Main findings
**Studies examining the outcomes of patients with drug‐related atrioventricular block in the absence of hyperkalemia or other abnormalities**							
Zeltser D., 2004	Retrospective, 169	78 ± 8.7, 60.4%	Second‐degree AVB: 79% Third‐degree AVB: 21%	Pts treated with Vaughan Williams class I/III antiarrhythmics AVB due to one of the following: ○ Acute myocardial infarction ○ Vasovagal syncope ○ Digitalis toxicity ○ Radiofrequency ablation	3 weeks	Ν/Α	Drug discontinuation: AVB resolved in 41% of pts within 48 h. AVB recurred in 56% of pts within 3‐week follow‐up. True drug‐induced AVB was rare: 15% of pts had AVB linked to meds (resolved after discontinuation, no recurrence in 3 weeks)
Kennebäck G., 2007	Prospective, 17	77 ± 7, 53%	High‐degree drug‐related AVB; all received permanent pacemaker at inclusion. Included if bradycardic/antiarrhythmic therapy could be stopped and AVB resolved by discharge or 1‐month follow‐up.	Heart valve surgery during the last year Permanent atrial fibrillation	2 years	9 ± 7 months	High‐grade AVB recurred in 59% of pts; higher recurrence in pts with QRS ≥ 120ms (75%) versus QRS < 120ms (20%) (*p* < 0.05). 35% of pts developed atrial tachyarrhythmias requiring antiarrhythmic drugs and backup pacing. Overall, 94% of pts required pacing.
Lee J.H., 2009	Retrospective, 38	68 ± 11, 44.7%	Drug‐related bradycardia:	Bradycardia due to one of the following:	18 ± 8 months	Ν/Α	Only one of seven pts presenting with drug‐related third‐degree AVB had true drug‐induced AVB.
			Sinus bradycardia (≤ 40 beats/min): 34.2% Sinus bradycardia with junctional escape beats: 47.4% Second‐degree AVB: 0% Third‐degree AVB: 18.4%	Acute myocardial infarction Antiarrhythmic drugs other than β‐blockers or non‐DHP CCBs Digitalis toxicity Electrolyte imbalances Failure of a previously implanted pacemaker device			
Osmonov D., 2012	Retrospective/Prospective, 668	68.2 ± 15.4, 49.6%	Type II second‐degree AVB Third‐degree AVB 2:1 AVB Atrial fibrillation with bradycardia (average heart rate ≤ 40 beats/min on 24h Holter monitoring)	Myocardial infarction Electrolyte abnormalities Digitalis toxicity Vasovagal syncope	1 week	N/A	Drug discontinuation: AVB resolved in 72% of pts within 72 h. AVB recurred in 27% of pts. Drug‐induced AVB: 52% AVB not attributed to meds: 48%
					N/A for patients in whom		
					AVB recurred after hospital discharge (delayed recurrence subgroup)		
Knudsen MB., 2013	Retrospective, 55	77 ± 10, 56%	Pts with drug‐related AVB treated with temporary pacemaker:	No ECG documentation Acute myocardial infarction Sick sinus syndrome Digitalis toxicity Renal failure in relation to treatment with sotalol or atenolol Severe electrolyte disturbance Vagal reaction Perioperative AVB Other causes not related to drugs	N/A	N/A	Drug discontinuation: 89% pts required permanent pacemaker implantation. Use of a temporary pacemaker: complications (infection or displacement) in 11% of cases.
					The mean follow‐up time for patients not requiring a permanent pacemaker was 1497 ± 1538 days.		
			Second‐degree Mobitz type II AVB: 18.2% Third‐degree AVB: 81.8% In the setting of AVB during atrial fibrillation, only pts with a prolonged pause greater than or equal to 5 s were included.				
Sayah S., 2016	Prospective, 49	65.8 ± 17.7, 53%	Second‐degree AVB: 20.4% Third‐degree AVB: 79.6%	Myocardial infarction Electrolyte abnormalities Digitalis toxicity Vasovagal syncope Class I/III antiarrhythmics	6 months	N/A	Drug discontinuation: 43% of pts had AVB recovery. AVB recurred in 50% of pts over 6‐month follow‐up; they were implanted with a permanent pacemaker.
Jordan‐Martinez L., 2020	Retrospective, 127	79 [71–83], 50.4%	Pts with drug‐related AVB: Third‐degree AVB 2:1 AVB Second‐degree Mobitz type II AVB Atrial fibrillation and AVB	Acute coronary syndrome Hyperkalemia ≥ 6.5 mEq/L Increased digoxin levels > 2 ng/mL AVB secondary to radiofrequency ablation Clinical inability to discontinue the medication	3 years	65 days [12–201 days]	Drug discontinuation: AVB resolved in 47.2% of pts within 48 h. AVB recurred in 66.6% of pts during the three‐year follow‐up period. Permanent pacemaker implantation: 84.36% AVB resolution without recurrence after drug discontinuation: 18.1%
							Factors associated with an increased overall need for permanent pacing:
							Heart rate < 35 bpm. Increased QRS duration. Clinical presentation other than syncope or presyncope.
							Factors associated with a lower overall need for permanent pacing:
							Combination of flecainide or amiodarone with β‐blockers or non‐DHP.
**Studies examining outcomes of drug‐related atrioventricular block in the presence or absence of hyperkalemia and other contributing abnormalities**							
Santos JG., 2024	Retrospective, 162	78 ± 8, 56%	Drug‐related AVB and/or hyperkalemia‐related AVB.	Acute coronary syndrome Pts who required a permanent pacemaker implantation to tolerate bradyarrhythmia‐inducing therapy	10 months [17], maximum follow‐up period: 130 months	N/A	Drug discontinuation and hyperkalemia correction: 82.1% of pts had persistent AVB requiring permanent pacemaker during initial admission AVB recovery: 17.9%; they were discharged without a permanent pacemaker. 82.8% of pts with initial AVB recovery had AVB relapse over 130‐month follow‐up. 97% of pts required permanent pacemaker; 3% had sustained AVB recovery during follow‐up
			2:1 AVB: 17.3% Second‐degree Mobitz type II AVB: 3.1% Complete AVB: 73.5% Atrial fibrillation and AVB: 6.2%				
							Factors linked to initial AVB recovery:
							CKD requiring dialysis Greater potassium levels Higher doses of bradycardic/antiarrhythmic medications Use of multiple bradycardic/antiarrhythmic drugs in combination Concurrent presence of both drug therapy and hyperkalemia
Duarte T., 2019	Retrospective, 153	82 ± 11, 47%	Drug‐related bradyarrhythmia and/or hyperkalemia‐related bradyarrhythmia Sinus bradycardia: 16% 2:1 AVB: 12% Complete AVB (including pts with atrial fibrillation and AVB): 63% Atrial fibrillation with slow ventricular response: 9%	N/A	24 ± 2 months*	N/A	Pts with drug‐related and/or hyperkalemia‐related AVB:
							50.4% received a permanent pacemaker during their initial hospitalization. 68.7% were implanted with a pacemaker by the end of the follow‐up period.
Habbout A., 2023	Retrospective, 93	76 ± 14, 49%	Pts with sinus node dysfunction or AVB and subsequent recovery of rhythm	Lack of medical records Not sinus node dysfunction or AVB Acute myocardial infarction Periprocedural sinus node dysfunction or AVB (surgery, percutaneous valve replacement, radiofrequency ablation) Death during the first hospitalization Implantation of a permanent pacemaker during the first hospitalization	800 days*	177 days (interquartile range: 15–470 days)*	Pts with AVB: 28% needed a pacemaker during the follow‐up period
			Sinus node dysfunction: sinus pauses > 3 s, sinus arrest, or sinoatrial exit block: 12% Second‐degree Mobitz type II AVB (including 2:1 AVB): 16% Third‐degree AVB: 72% Reversible etiology (drugs, hyperkalemia, acute kidney injury, vasovagal reaction, infection, hypothermia)				

Abbreviations: CKD, chronic kidney disease; N/A, nonavailable; pts, patients.

* These figures refer to each study's entire cohort, which included patients with drug‐related bradycardia in general and not exclusively patients with AVB.

### 2.1 Studies Examining the Outcomes of Patients With Drug‐Related Atrioventricular Block in the Absence of Hyperkalemia or Other Abnormalities

Zeltser et al. were the first to show that AVB is commonly associated with β‐blocker and/or non‐DHP CCB therapy, but drug therapy is rarely the underlying cause [1]. Specifically, the study population consisted of 169 patients with second‐ or third‐degree AVB, most of whom were elderly with hypertension and/or structural heart disease. Of these patients, 54% had drug‐related AVB, whereas the remainder presented with AVB in the absence of drug therapy. Among the patients with drug‐related AVB, the majority were on β‐blockers (67.4%), while 32.6% were on non‐DHP CCBs, and 14.1% were on medications from both drug classes [1]. According to electrocardiographic characteristics, most patients had infranodal block, with only a minority having AV nodal block. Upon hospitalization for AVB, drug therapy was discontinued in 86% of patients with drug‐related AVB. Drug discontinuation was followed by a spontaneous resolution of AVB within 48 h in 41% of these patients. However, 56% of them experienced a recurrence of AVB within the follow‐up period of 3 weeks, often of a worse degree, leading to syncope [1]. Interestingly, spontaneous resolution of AVB was also observed in 23% of patients who had AVB in the absence of drug therapy. Although AVB resolution was less common in this setting, it could be concluded that improvement in AV conduction upon cessation of medications is often coincidental. Challenging the prevailing belief, this study demonstrated that true drug‐induced AVB is rare, as only 15% of patients with drug‐related AVB had AVB directly attributable to the medications, defined as AVB that resolved after drug discontinuation and did not recur during the 3‐week follow‐up period [1]. Furthermore, it was shown that AV nodal block during drug therapy is a poor predictor of causation.

In another study, Kennebäck et al. suggested that patients on β‐blockers who develop high‐degree AVB and have a QRS duration ≥ 120 ms should be implanted with a permanent pacemaker without further investigation or observation [7]. This study examined patients with symptomatic high‐degree drug‐related AVB who were implanted with a permanent pacemaker at presentation and were enrolled only if their AVB resolved following withdrawal of the offending medication during hospitalization or by the first follow‐up at 1 month [7]. Most individuals were elderly with hypertension and/or ischemic heart disease, as well as heart failure. Furthermore, most patients were on β‐blockers (88%), with the remainder receiving sotalol, verapamil, or digoxin (used alone or in combination). Over a 2‐year period, high‐grade AVB recurred in 59% of patients, with a significantly higher recurrence rate in those with increased QRS duration (≥ 120 ms) (75%) compared to those with normal QRS duration (< 120 ms) (20%) (*p* < 0.05) [7]. The mean time from inclusion to documented high‐degree AVB was 9 ± 7 months. Additionally, 35% of the patients developed symptomatic atrial tachyarrhythmias necessitating antiarrhythmic drug treatment and backup pacing. Consequently, 94% of the total patients required permanent pacing due to either high‐degree AVB recurrence or atrial tachyarrhythmias requiring antiarrhythmic drug treatment and concomitant backup pacing [7].

Consistent with the findings of Zeltser et al., Lee et al. reported that only one out of seven patients presenting with third‐degree AVB and concomitant β‐blocker and/or non‐DHP CCB therapy had a true drug‐induced AVB attributable to these medications [8]. The mean follow‐up duration in this study was 18 ± 8 months, but this refers to the entire study cohort, which included patients with drug‐related bradycardia in general, not exclusively patients with AVB. Moreover, Osmonov et al. reported a higher incidence of AVB attributable to the medications as compared to the findings of Zeltser et al., as well as higher rates of resolution and lower rates of recurrence of AVB following drug discontinuation [9]. However, pacemaker implantation was still required in 48% of patients with drug‐related AVB [9]. Specifically, this study included 668 patients with symptomatic type II second‐ or third‐degree AVB, 2:1 AVB, and atrial fibrillation with bradycardia (average heart rate ≤ 40 beats/min on 24‐h Holter monitoring). Most of them were elderly and had comorbidities such as hypertension, congestive heart failure, coronary artery disease, and diabetes mellitus. Among the study patients, 108 (16.2%) had drug‐related AVB [9]. The majority were on β‐blockers (69.4%), followed by digoxin (36.1%), non‐DHP CCBs (15.7%), and class Ic or III antiarrhythmics (5.5%). Additionally, 26.9% were on a combination of multiple bradycardic or antiarrhythmic medications, and 20.4% were taking a combination of β‐blockers and digoxin. Drug therapy was discontinued in all patients with drug‐related AVB, and spontaneous resolution of AVB within 72 h occurred in 72% of these patients [9]. Remarkably, 27% experienced a recurrence of AVB despite discontinuation of the offending drugs. Overall, 52% of the patients with drug‐related AVB had AVB attributed to the medications, defined as AVB that resolved after drug discontinuation and did not recur during the 1‐week follow‐up period. Meanwhile, 48% had AVB not attributed to the medications and required permanent pacemaker implantation. Notably, the follow‐up period is not reported for patients in whom AVB recurred after hospital discharge (delayed recurrence subgroup). Interestingly, the presence of atrial fibrillation with bradycardia, as well as the use of digoxin, the combination of digoxin and b‐blockers, and the use of any drug combination, were significantly more frequent in the group of patients with AVB attributed to drug therapy [9]. Furthermore, according to electrocardiographic characteristics, infranodal AVB was significantly more frequent in the group of patients with AVB not attributed to drug therapy, while the group of patients with AVB attributed to drug therapy had a higher prevalence of AVB at the level of the AV node [9].

Of note, Knudsen et al. demonstrated that the use of a temporary pacemaker combined with drug discontinuation in patients with drug‐related AVB was associated with a high complication rate, while the need for a permanent pacemaker was substantial, suggesting an early move to permanent pacing in such cases [10]. The study population consisted of 55 patients with second‐degree Mobitz type II AVB or third‐degree AVB, including those with atrial fibrillation and AVB, with only patients exhibiting a prolonged pause of ≥ 5 s being included. Most of the patients were predominantly elderly and had underlying heart disease. Furthermore, most of them were on β‐blockers (49%), with the remainder on digoxin (5%), sotalol (4%), or a combination of multiple drug classes (42%) [10]. Despite drug discontinuation, 89% of the patients ultimately required permanent pacemaker implantation, with 47 having an indication during their index hospitalization and 2 following readmissions due to AVB relapse [10]. The mean follow‐up time for patients not requiring a permanent pacemaker was 1497 ± 1538 days. Significantly, insertion of a temporary electrode resulted in complications (infection or displacement) in 11% of cases, suggesting that immediate implantation of a permanent pacemaker in patients with drug‐related AVB may be a reasonable approach when temporary pacing is required. Another noteworthy finding was that neither the degree of AVB, the QRS duration, nor any other variable was a significant predictor of permanent pacemaker implantation [10].

In the same line as the previous studies, Sayah et al. demonstrated that drug‐related AVB exhibits a significant rate of recurrence despite initial resolution [11]. This study included 49 patients with symptomatic second‐ or third‐degree AVB, most of whom were elderly. The study patients were divided into two groups: those who developed AVB without receiving drugs affecting AV conduction (28 patients) and those with drug‐related AVB (21 patients). Among the patients with drug‐related AVB, most were on β‐blockers (81%), and the rest were on CCBs (9.5%), or medications from both drug classes (9.5%) [11]. The results indicated that 43% of the patients with drug‐related AVB had AVB recovery after discontinuing the offending agents. However, excluding one case lost to follow‐up, 50% of them required permanent pacemaker implantation due to AVB recurrence over a 6‐month follow‐up period [11].

In a recent study, Jordan‐Martinez et al. analyzed patients with drug‐related AVB, focusing on identifying predictive factors for pacemaker need [5]. The study population consisted of 127 patients with third‐degree AVB, 2:1 AVB, or second‐degree Mobitz type II AVB, including individuals with atrial fibrillation and AVB. Most of the study patients were elderly and had comorbidities such as hypertension, diabetes mellitus, atrial fibrillation, and ischemic or other heart disease. Also, most patients were on β‐blockers (78%), 18.1% were on non‐DHP CCBs, and 11.8% were on digoxin [5]. Additionally, 3.9% and 4.8% were receiving flecainide or amiodarone, respectively, in combination with the previously mentioned drugs. Furthermore, 8.6% of the patients were on a combination of digoxin, flecainide, or amiodarone with either β‐blockers or non‐DHP CCBs [5]. In keeping with the studies mentioned above, drug discontinuation led to the spontaneous resolution of AVB within 48 h in 47.2% of patients. However, 66.6% of these patients experienced a recurrence of AVB during the 3‐year follow‐up period (median time to AVB recurrence: 65 days; interquartile range: 12–201 days) [5]. Overall, 84.36% of patients required permanent pacemaker implantation in the acute setting or during follow‐up, while only 18.1% experienced AVB resolution without recurrence after drug discontinuation [5]. Importantly, this study identified several factors associated with the overall need for permanent pacing. These included a heart rate < 35 bpm (odds ratio [OR] = 8.12; 95% confidence interval [CI] 1.82–36.17; *p* = 0.006), increased QRS duration (OR = 5.65; 95% CI = 1.77–18.04; *p* = 0.003), and a clinical presentation other than syncope or presyncope (OR = 4.09; 95% CI = 1.18–14.13; *p* = 0.026). Interestingly, a combination of flecainide or amiodarone with β‐blockers or non‐DHP CCBs was associated with a significantly lower need for pacemaker implantation (OR = 0.12; 95% CI 0.02–0.66; *p* = 0.014) [5].

### 2.2 Studies Examining Outcomes of Drug‐Related Atrioventricular Block in the Presence or Absence of Hyperkalemia and Other Contributing Abnormalities

In contrast to the previously mentioned studies that excluded patients with hyperkalemia, Santos et al. expanded the scope of the research by including patients with drug‐related AVB, hyperkalemia‐related AVB, or both conditions [12]. Specifically, this study involved 162 individuals with 2:1 AVB, second‐degree Mobitz type II AVB, and complete AVB, including patients with atrial fibrillation and AVB. Most participants were elderly and had comorbidities such as hypertension, dyslipidemia, diabetes mellitus, ischemic heart disease, and chronic kidney disease, including a small proportion of patients on dialysis. Of these, 88.9% had drug‐related AVB, 21% had hyperkalemia‐related AVB, and 10.5% presented with both conditions [12]. Additionally, 74.7% of the patients were on β‐blockers, followed by 6.8% on non‐DHP CCBs, 5.6% on digoxin, 7.4% on class Ic or III antiarrhythmics, and 4.9% on multiple bradycardic or antiarrhythmic medications [12]. Following drug discontinuation and correction of hyperkalemia, 82.1% of patients had persistent AVB requiring permanent pacemaker implantation during their initial admission, while the remainder (17.9%) recovered from AVB and were discharged without a permanent pacemaker [12]. Remarkably, among patients with initial AVB recovery, 82.8% experienced an AVB relapse during a maximum follow‐up of 130 months (median follow‐up: 10 months; interquartile range: 17 months). Overall, 97% of the patients required permanent pacemaker implantation, while only 3% had sustained AVB recovery during the follow‐up period. Factors associated with an initial AVB recovery included the presence of chronic kidney disease requiring dialysis (OR = 7.6; 95% CI = 1.2–47.5; *p* = 0.03), greater potassium levels (OR = 2.3; 95% CI = 1.28–4.0; *p* < 0.01), higher doses of bradycardic/antiarrhythmic medications (OR = 2.2; 95% CI = 1.13–4.4; *p* = 0.02), the use of multiple bradycardic/antiarrhythmic drugs in combination (OR = 9.0; 95% CI = 2.02–40.3; *p* < 0.01), and the concurrent presence of both drug therapy and hyperkalemia (OR = 5.2; 95% CI = 1.8–15.1; *p* < 0.01) [12]. However, AVB recurrence rates were exceedingly high in all these patient subsets, even in those receiving the highest dosage or combinations of multiple AV nodal blocking agents [12]. Additionally, the inclusion of patients with hyperkalemia‐related AVB did not result in a lower need for permanent pacing, nor did it increase sustained recovery compared to studies that focused solely on patients with drug‐related AVB. In fact, the opposite was observed; namely, the requirement for permanent pacing was higher, and sustained recovery was lower, suggesting that hyperkalemia, as an additional provoking factor, may have led to the identification of more patients with underlying conduction disease. An alternative explanation may be that patients with hyperkalemia represent an older and sicker population, often burdened with advanced chronic kidney disease, heart failure, and polypharmacy. The use of medications affecting sinus node or atrioventricular conduction (e.g., antidepressants, anticonvulsants) may further contribute to persistent conduction disturbances in this vulnerable group. Finally, it was demonstrated that the absence of conduction abnormalities in the baseline ECG (retrieved from electronic health records before the index admission) was not associated with AVB recovery following drug discontinuation or hyperkalemia correction, nor with sustained long‐term rhythm recovery [12].

In a similar study that included elderly patients with various bradyarrhythmias, including sinus bradycardia and atrial fibrillation with slow ventricular response, Duarte et al. reported that, among the subset with drug‐related and/or hyperkalemia‐related AVB, 50.4% received a permanent pacemaker during their initial hospitalization, and 68.7% had a pacemaker implanted by the end of follow‐up. The reported mean follow‐up duration was 24 ± 2 months, but this refers to the entire study cohort and not exclusively to patients with AVB [13]. Even further, Habbout et al. studied elderly patients (mean age 76 ± 14) with sinus node dysfunction or second‐ or third‐degree AVB who experienced rhythm recovery after addressing reversible etiologies which included not only bradycardic or antiarrhythmic medications and hyperkalemia but also acute kidney injury, vasovagal reactions, infections, and hypothermia [14]. Among the subset of patients with AVB, 28% required a pacemaker. During a maximum follow‐up of 800 days, the reported mean time to readmission was 177 days (interquartile range: 15–470 days), but both figures refer to the entire study cohort, which included patients with drug‐related bradycardia in general and not exclusively patients with AVB. Notably, the number and severity of comorbidities in this study were comparable to those reported in previous studies, with a high prevalence of hypertension (63%), diabetes (26%), chronic kidney disease (23%), and ischemic heart disease (18%). Although the pacemaker implantation rate in this study was lower than those reported by Santos et al. and Duarte et al., this may be attributed to the inclusion of patients with a wider spectrum of potentially acute and reversible causes of bradycardia and hyperkalemia, increasing the likelihood of identifying a transient, causative factor and reducing the need for permanent pacing.

## 3 Critical Analysis of Current Evidence and Management of Patients With Drug‐Related Atrioventricular Block

Current evidence suggests that drug‐related AVB is a common clinical problem in the elderly [1, 5, 7, 8, 9, 10, 11, 12, 13, 14]. However, true drug‐induced AVB seems to be rare [1, 5, 12]. Despite the initial resolution of AVB following drug discontinuation, it is convincingly reported that the rates of recurrence and the necessity for permanent pacing are remarkably high [1, 5, 7, 8, 9, 10, 11, 12, 13, 14]. This trend appears to be evident even in patients receiving high doses of bradycardic or antiarrhythmic medications, those on drug combinations, and those with additional reversible factors such as hyperkalemia [12]. Nevertheless, drugs, metabolic disturbances, or a combination of them seem to unmask the presence of severe underlying conduction system disease, indicating that early intervention with permanent pacing may be advisable.

Β‐blockers and non‐DHP CCBs exert their negative dromotropic effects primarily at the level of the AV node [15, 16]. Neither β‐blockers nor non‐DHP CCBs significantly affect conduction in the His‐Purkinje system [17, 18]. Similarly, digoxin slows conduction in the AV node but, at therapeutic levels, has minimal effects on the His‐Purkinje system [19, 20]. Bearing in mind that these medications primarily slow the sinus rate and conduction at the AV node, they are expected to prevent, not provoke, conduction block in the more distal (infranodal) parts of the conduction system. Therefore, when second‐ or third‐degree AVB occurs during therapy with β‐blockers, non‐DHP CCBs, or digoxin, it suggests that the underlying infranodal conduction disease is likely severe and may eventually progress to a permanent disturbance. Furthermore, patients with intermittent infrahisian block may also present with concomitant typical nodal AVB, which can be influenced by a transient, contributing factor and may resolve following its discontinuation. As is very well known, complete AVB does not necessarily imply an infranodal origin, and it is often difficult to distinguish nodal from infranodal AVB using surface ECG alone, particularly when these conditions coexist [21]. Additionally, infranodal Wenckebach block may also be encountered. Moreover, in cases of nodal AVB, progression to infranodal disease may occur over time. The timeline of AVB recurrence after apparent resolution could help inform clinical decision‐making. However, the available studies have varying follow‐up durations, and only two have reported recurrence intervals, with a median of 65 days in one and a mean of 9 months in the other. These findings suggest that AVB tends to recur within months after resolution following drug discontinuation, but more data are needed to clearly define its recurrence patterns and natural history.

Notably, class Ic antiarrhythmics prolong the His‐ventricular (HV) interval, particularly when underlying infranodal conduction disease is present. Specifically, flecainide is often utilized as a drug challenge during electrophysiological studies to reveal His‐Purkinje disease in cases with a wide QRS complex, syncope, and a normal HV interval, as evidenced by a drug‐induced increase in the HV interval to more than 100 ms [22]. Similarly, class III antiarrhythmics can also prolong the HV interval and reveal latent conduction system pathology [23]. As far as hyperkalemia is concerned, a biphasic response has been described. Mild hyperkalemia lowers the resting membrane potential (making it less negative) more than it reduces the threshold potential. This diminishes their difference, leading to increased cellular excitability and enhanced conduction [24, 25]. More severe hyperkalemia further lowers the resting membrane potential, reducing the percentage of available sodium channels and decreasing the inward sodium current during phase 0 of the action potential, which leads to slower conduction velocity and increased HV interval. Consequently, hyperkalemia may unmask the presence of severe underlying conduction system disease.

Only a limited number of studies have examined and identified predictive factors for the overall need for permanent pacing, initial recovery from drug‐related and/or hyperkalemia‐related AVB, and recurrence following drug discontinuation and/or correction of hyperkalemia, often with conflicting findings [1, 5, 7, 9, 10, 12]. As previously mentioned, Jordan‐Martinez et al. demonstrated that in patients with drug‐related AVB, a heart rate < 35 bpm and presenting symptoms other than syncope or presyncope were associated with a higher need for permanent pacing [5]. However, other studies have not consistently confirmed these findings. Regarding the role of QRS duration, Jordan‐Martinez et al. reported that an increased QRS duration was associated with a greater need for permanent pacing in patients with drug‐related AVB [5]. In the same line, Kennebäck et al. suggested higher recurrence rates in patients with drug‐related AVB who had an increased QRS duration compared to those with normal QRS duration [7]. On the contrary, Knudsen et al. [10] did not demonstrate such an association, while Santos et al. [12] reported that the absence of conduction abnormalities on baseline ECGs in patients with drug‐related and/or hyperkalemia‐related AVB was not associated with AVB recovery following drug discontinuation or hyperkalemia correction, nor did it predict sustained long‐term rhythm recovery.

The available evidence regarding the role of AV block level based on electrocardiographic characteristics is also inconsistent. Zeltser et al. suggested that AV nodal block during drug therapy is a poor predictor of causation [1]. Conversely, Osmonov et al. showed that infranodal AVB was significantly more frequent in the group of patients with AVB not attributed to drug therapy, while the group of patients with AVB attributed to drug therapy had a higher prevalence of AVB at the level of the AV node [9].

Concerning the role of drug combinations, Jordan‐Martinez et al. reported that the combination of flecainide or amiodarone with β‐blockers or non‐DHP CCBs was linked to a significantly lower need for pacemaker implantation [5]. In keeping with these findings, Osmonov et al. demonstrated that the use of drug combinations was significantly more frequent in the group of patients with AVB attributed to drug therapy [9]. On the other hand, Santos et al. demonstrated that, although combinations of multiple bradycardic or antiarrhythmic drugs increased the likelihood of initial AVB resolution in patients with drug‐related and/or hyperkalemia‐related AVB, the recurrence rates of AVB were exceedingly high [12]. Concerning the role of higher versus lower doses of bradycardic/antiarrhythmic drugs, it is traditionally believed that higher doses of these agents may induce AVB in a straightforward cause‐and‐effect manner. However, the limited available evidence contradicts this common assumption. As was previously mentioned, Santos et al. demonstrated that, although higher doses are associated with higher rates of initial AVB resolution after drug discontinuation and/or hyperkalemia correction, the recurrence rates of AVB and the need for permanent pacing remain very high in these patients [12]. This study also indicated that this pattern applies to patients with chronic kidney disease requiring dialysis, those with greater potassium levels, and those with a combination of drug therapy and hyperkalemia, who similarly show higher initial recovery rates but persistently high recurrence rates and eventual permanent pacing requirements [12].

It should be stressed that all available studies primarily involved elderly patients with common comorbidities, representing frail populations. Thus, extrapolating these findings to younger, healthier populations would be inappropriate. Furthermore, the current body of evidence is derived predominantly from retrospective observational studies, which inherently suffer from significant limitations and potential biases. One of the most notable concerns is selection bias, as these studies primarily focus on patients with symptomatic or electrocardiographically detected bradyarrhythmias who were admitted to specialized cardiac units. However, many patients with mild or transient bradyarrhythmias may have been managed in emergency rooms or geriatric/internal medicine settings without formal cardiology evaluation or hospitalization. This may have led to a systematic underrepresentation of a broader patient population, limiting the generalizability of findings, and potentially skewing recurrence and pacemaker implantation rates toward more severe cases.

Another major limitation is the absence of electrophysiologic (EP) data at the time of AVB presentation, as the site and mechanism of AVB may have been significantly misidentified when assessed by surface ECG alone. This methodological pitfall may help explain the conflicting observations regarding the causal role of AVB level in determining whether AVB is drug‐related. Indeed, as mentioned earlier, it is difficult to attribute β‐blockers or non‐DHP CCBs as the cause of infranodal AVB, raising the possibility that some cases labeled as drug‐related AVB may instead represent intermittent conduction disease or ECG misinterpretation.

Additionally, most studies did not measure plasma concentrations of drugs acting on the His‐Purkinje system, which is a critical limitation in elderly patients. Low body mass index (BMI), chronic kidney disease, and polypharmacy can significantly alter drug metabolism and accumulation, potentially exacerbating infranodal conduction abnormalities. Without plasma drug level monitoring, establishing a causal relationship between these medications and AVB is challenging, making any conclusions about their direct role in AVB speculative.

For all these reasons, substantial uncertainty remains in identifying patients at early risk of AVB recurrence. It is therefore not surprising that reliable risk scores for predicting recurrence after initial AVB resolution are still lacking. This gap in clinical tools makes it challenging to accurately assess patients at higher risk of AVB relapse. Addressing these gaps through prospective studies with standardized EP assessment and drug level monitoring is crucial to improving diagnostic accuracy and clinical decision‐making in drug‐related AVB.

**Figure 1:**
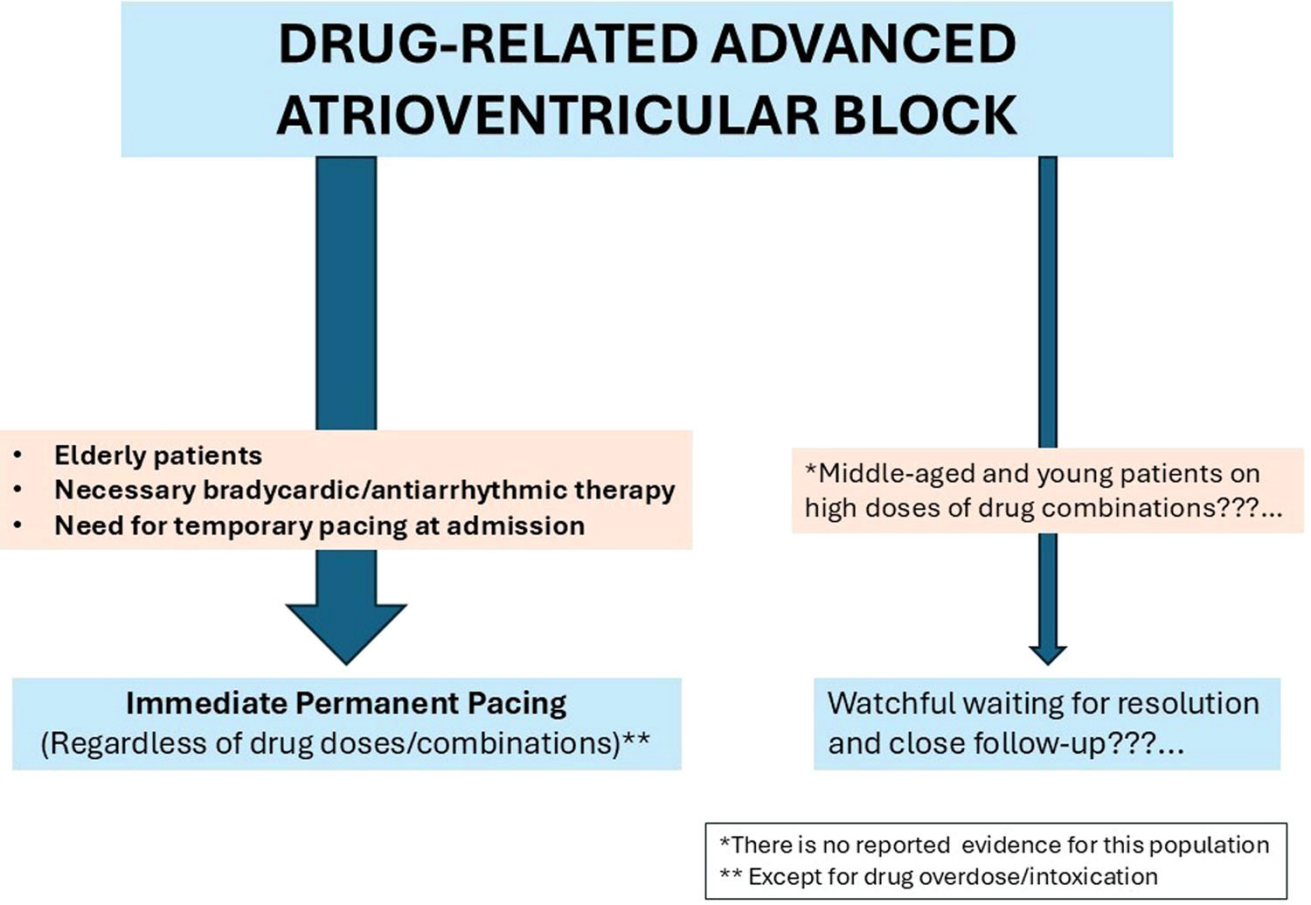
Schematic representation of the management pathway for drug‐related advanced atrioventricular block.

In this context of limited evidence, managing patients with drug‐related AVB is challenging. Given that true drug‐induced AVB is rare and recurrence rates are high, immediate permanent pacemaker implantation may be advisable, especially for frail elderly patients with drug‐related AVB (Figure 1). This strategy may be relevant even for patients on high doses of bradycardic or antiarrhythmic drugs, those on drug combinations, and those with additional reversible factors like hyperkalemia. Moreover, considering the notably high need for permanent pacing in patients with drug‐related AV block and atrial tachyarrhythmias when antiarrhythmic treatment is necessary, adopting an early permanent pacing approach seems prudent (Figure 1). Finally, because temporary pacing carries a high risk of complications, transitioning directly to permanent pacemaker implantation should be prioritized when temporary pacing is indicated (Figure 1). There are no data in the literature regarding drug‐related AVB in younger populations. It would be reasonable to assume that a more conservative strategy, namely watchful waiting for AVB resolution and close follow‐up could be adopted in younger individuals, particularly in the setting of high doses of bradycardic or antiarrhythmic medications. However, this assumption remains speculative and should be examined in future studies (Figure 1).

## 4 Conclusions

Drug‐related AVB represents a common clinical problem in the elderly, even though true drug‐induced AVB is rare. Despite initial recovery after discontinuing the offending medications, recurrence rates are notably high, very often leading to permanent pacemaker implantation. This trend seems to hold even for patients on high doses of bradycardic or antiarrhythmic drugs, those on drug combinations, and those with additional reversible factors such as hyperkalemia. Based on these findings, early consideration for permanent pacemaker implantation may be a prudent strategy in all frail elderly patients with drug‐related AVB. Moreover, given the high need for backup pacing in patients with drug‐related AV block and atrial tachyarrhythmias when antiarrhythmic treatment is necessary, an early permanent pacing approach is recommended. Additionally, in cases where temporary pacing is necessary, immediate placement of a permanent pacemaker may reduce complications and improve clinical outcomes. Undoubtedly, further research may identify reliable predictors that can guide clinical decision‐making in the setting of drug‐related and/or hyperkalemia‐related AVB.

## Conflicts of Interest

The authors declare no conflicts of interest.

## Supporting information

Table 2 30‐03‐25.

## Data Availability

Data sharing does not apply to this article as no new data were created or analyzed in this study.
