# Supplementary material for: Clinical Significance and Management of Atrioventricular Block Associated With Bradycardic/Antiarrhythmic Drug Therapy: Drug‐Induced or Drug‐Revealed?
Source: J Cardiovasc Electrophysiol. 2025 Apr 28;36(7):1643–53. doi: 10.1111/jce.16697 (PMC12246518; doi:10.1111/jce.16697)
Supplement: Supplementary file 1 — Table 2 30‐03‐25. [file JCE-36-1643-s001.docx]

| **First author, year** | **Bradycardic / antiarrhythmic drugs / other reversible factors** | **Significant comorbidities** | **QRS characteristics** |
| --- | --- | --- | --- |
| **Studies examining the outcomes of patients with drug-related atrioventricular block in the absence of hyperkalemia or other abnormalities** | | | |
| Zeltser D.,  2004 | - AVB in the absence of drugs that affect AV conduction: 46% - Drug-related AVB: 56%   - Vaughan Williams classes II and IV   - β-blockers: 67.4%   - non-DHP CCBs: 32.6%   - drugs from both classes: 14.1% | - IHD: 39.1% - Htn: 58% - Other heart diseases: 8.9% - No heart disease: 21.3% | QRS duration: 124 ± 27 ms |
| Kennebäck G., 2007 | Vaughan Williams classes I-IV and digoxin   - β-blockers: 88% - Remaining pts on sotalol, verapamil, or digoxin (alone or in combination) | - Htn: 6/17 - IHD: 4/17 - Htn and IHD: 2/17 - HF: 3/17 - Suspicion of AF: 1/17 - Tremor: 1/17 | QRS duration: 130 ± 32 ms   - Normal QRS duration: 5 pts - Increased QRS duration (≥120 ms): 12 pts   - RBBB: 2 pts   - IVCD: 1 pt   - Bifascicular block: 9 pts |
| Lee J.H., 2009 | Vaughan Williams classes II and IV   - β-blockers: 89.5% - non-DHP CCBs: 23.7% - drugs from both drug classes: 13.2% | - Htn: 76.3% - IHD: 55.3% - DM: 31.6% | QRS duration: 97.9 ± 17.3 ms |
| Osmonov D., 2012 | - AVB in the absence of drugs that affect AV conduction: 83.8% - Drug-related AVB: 16.2%   Vaughan Williams classes I-IV   - - β-blockers: 69.4%   - digoxin: 36.1%   - non-DHP CCBs: 15.7%   - class Ic/III antiarrhythmics: 5.5%   - combinations of multiple bradycardic/antiarrhythmic medications: 26.9%   - combinations of β-blockers and digoxin: 20.4% | N/A for the entire cohort  For pts with drug-related AVB:   - Htn: 83.3% - Coronary artery disease: 50% - Congestive HF: 38% - DM: 36.1% | Ν/Α |
| Knudsen MB., 2013 | Vaughan Williams classes II-IV and digoxin   - β-blockers: 49% - digoxin: 5% - sotalol: 4% - combinations of multiple drug classes: 42% | - IHD: 25% - Dilated cardiomyopathy: 4% - Aortic valve stenosis: 7% - Paroxysmal AF or AFL: 20% - Chronic AF or AFL: 40% - Reduced LVEF: 25% - No heart disease: 27% | QRS duration: 118 ± 27  Type of BBB:   - LBBB: 15% - RBBB: 9% - RBBB + LAFB: 9% - RBBB + LPFB: 4% - Undetermined: 5% - No BBB: 58% |
| Sayah S., 2016 | - AVB in the absence of drugs that affect AV conduction: 28 pts - Drug-related AVB: 21 pts   Vaughan Williams classes II and IV   - - β-blockers: 81%   - non-DHP CCBs: 9.5%   - drugs from both drug classes: 9.5% | N/A | 44.9% of pts with QRS duration > 120 ms |
| Jordan-Martinez L., 2020 | Vaughan Williams classes II, IV and digoxin. Pts receiving class Ic/III antiarrhythmics in addition to the previously mentioned drugs were also included.   - β-blockers: 78% - non-DHP CCBs: 18.1% - digoxin: 11.8% - 3.9% and 4.8% were receiving flecainide or amiodarone, respectively, in combination with the previously mentioned drugs - 8.6% of the pts were on a combination of digoxin, flecainide, or amiodarone with either β-blockers or non-DHP CCBs. | - Htn: 85% - IHD: 28.3% - Other heart disease: 20.5% - DM: 55.1% - Atrial fibrillation 32.3% | 120 [110-140] |
| **Studies examining outcomes of drug-related atrioventricular block in the presence or absence of hyperkalemia and other contributing abnormalities** | | | |
| Santos JG., 2024 | - Drug-related AVB: 88.9% - Hyperkalemia-related AVB: 21% - Drug-related AVB and hyperkalemia-related AVB: 10.5%   Vaughan Williams classes I-IV and digoxin   - β-blockers: 74.7% - non-DHP CCBs: 6.8% - digoxin: 5.6% - class Ic/III antiarrhythmics: 7.4%   - Amiodarone: 4.9%   - Sotalol: 1.2%   - Propafenone: 1.2% - combinations of medications from multiple drug classes: 4.9% | - Htn: 91% - Dyslipidemia: 51% - DM: 42% - IHD: 17% - CKD: 20% (3.1% of which on dialysis) | Prior ECG obtained: 69.8% of the pts  Pts with conduction abnormalities: 81.3%   - AV conduction abnormalities 33.9%   - 1st degree AVB: 29.5%   - 2nd degree Mobitz type I AVB: 4.5% - IV conduction abnormalities 69.8%   - LBBB: 23.9%   - RBBB: 16.8%   - LAFB: 8%   - RBBB + LAFB: 9.5%   - RBBB + LPFB: 0.9%   - Non-specific IV conduction abnormalities: 0.9% - AV and IV conduction abnormalities: 20.5% - Trifascicular block: 8.9% |
| Duarte T., 2019 | - Drug-related AVB: 85% - Hyperkalemia-related AVB: 3% - Drug-related and hyperkalemia-related AVB: 12%   In pts with solely drug-related bradyarrhythmia:  Vaughan Williams classes II-IV and digoxin   - β-blockers: 38% - non-DHP CCBs: 12% - amiodarone: 15% - digoxin: 5% - sotalol: 2% - propafenone: 3% - combinations of multiple drug classes: 25%   In pts with concomitant drug-related and hyperkalemia-related bradyarrhythmia: N/A | - IHD: 26% - HF: 9% - Htn: 92% - DM: 29% - Dyslipidemia: 41% - Renal failure: 19% | N/A |
| Habbout A., 2023 | Reversible etiology:   - Drug-related etiology   - β-blockers: 39%   - Non-DHP CCBs: 8%   - Ivabradine: 0%   - Digoxin: 4%   - Antiarrhythmics (amiodarone, flecainide): 2% - Isolated hyperkalemia: 8% - Hyperkalemia + drugs: 6% - Hyperkalemia + drugs + AKI: 8% - Hyperkalemia + AKI: 6% - AKI + drugs: 10% - Vagal cause: 6% - Other causes: 4% | - Htn: 63% - DM: 26% - Hypercholesterolemia: 24% - Dysthyroidism: 12% - Atrial fibrillation/Flutter: 44% - IHD: 18% - PAD: 6% - Previous stroke: 13% - CKD: 23% - Lung disease: 15% - Cancer: 32% - Autoimmune disease: 6% | N/A |

**Supplementary Table**: Studies examining the outcomes of patients with drug-related AV block (AVB). N/A: non-available; pts: patients; htn: hypertension; IHD: ischemic heart disease; PAD: peripheral arterial disease; HF: heart failure; DM: diabetes mellitus; CKD: Chronic kidney disease. Age is presented as mean ± SD or median [IQR].
